# Strain Investigation on Spin-Dependent Transport Properties of γ-Graphyne Nanoribbon Between Gold Electrodes

**DOI:** 10.1186/s11671-020-03461-3

**Published:** 2021-01-06

**Authors:** Yun Li, Xiaobo Li, Shidong Zhang, Liemao Cao, Fangping Ouyang, Mengqiu Long

**Affiliations:** 1 Hunan Key Laboratory of Super Micro-structure and Ultrafast Process, School of Physics and Electronics, Central South University, Changsha, 410083 China; 2 Department of Applied Physics, Hunan University of Technology and Business, Changsha, 410205 China; 3 Key Laboratory of Hunan Province for Statistical Learning and Intelligent Computation, Hunan University of Technology and Business, Changsha, 410205 Hunan China; 4 Science, Math and Technology, Singapore University of Technology and Design (SUTD), 8 Somapah Road, Singapore, 487372 Singapore; 5 Institute of Low-Dimensional Quantum Materials and Devices, School of Physical Science and Technology, Xinjiang University, Ürümqi, 830046 China

**Keywords:** γ-Graphyne nanoribbon, Strain engineering, Spin splitting, Electronic device, Density functional theory

## Abstract

Strain engineering has become one of the effective methods to tune the electronic structures of materials, which can be introduced into the molecular junction to induce some unique physical effects. The various γ-graphyne nanoribbons (γ-GYNRs) embedded between gold (Au) electrodes with strain controlling have been designed, involving the calculation of the spin-dependent transport properties by employing the density functional theory. Our calculated results exhibit that the presence of strain has a great effect on transport properties of molecular junctions, which can obviously enhance the coupling between the γ-GYNR and Au electrodes. We find that the current flowing through the strained nanojunction is larger than that of the unstrained one. What is more, the length and strained shape of the γ-GYNR serves as the important factors which affect the transport properties of molecular junctions. Simultaneously, the phenomenon of spin-splitting occurs after introducing strain into nanojunction, implying that strain engineering may be a new means to regulate the electron spin. Our work can provide theoretical basis for designing of high performance graphyne-based devices in the future.

## Introduction

Charge and spin are two main intrinsic properties of electron [1–3]. The traditional microelectronics often concentrate on the charge characteristic of electron, regardless of the spin states on the electron. And introducing the electric field [4, 5] to regulate the electrons transport of the semiconductor materials to realize the information transportation or processing has become a common method. With the continuous improvement of science and technology, the experiment of large integrated circuit is getting more and more than before [6]. The high density components of electronic and miniaturization has become an urgent need. In recent decades, scientists have begun to explore the spin characteristics of electron into molecular devices on the spintronics [7, 8]. The relaxation time of the spin is relatively long, which is not easily affected by the defects and impurities of the spin device, and can be achieved by a series of means, such as electric field, magnetic field and so on [9]. Therefore, a lot of modulating methods with respect to the spintronic properties of molecular junctions have become the focus of intensive research.

Compared with chemical doping [10–12] and electromagnetic field controlling [13, 14], strain engineering [15–17] is considered to be the most effective and controllable technique for nanomaterials. The interaction between the lattice and electron (spin, orbit, etc*.*) influence the electrical, magnetic, or optical characteristics of the material induced by strain engineering, which can lead to the emergence of other unique physical or chemical effects [18, 19]. What is more, strain is inevitably in the process of experimental sample preparation, which can be applied by different channels. For instance, the substrate is not prepared smoothly [20], the lattice parameters of the sample and the substrate material are not matched [21], or the crimp exists at the edge of the nanoribbons [22] and so forth.

Further, it was reported that strain has an obvious effect on the electronic structure of two-dimensional (2D) materials [23, 24]. When the uniaxial strain is applied, the shift of Dirac-cone of graphene can be observed [25].And the uniaxial strain in a biggish range can change the zero-band gap of graphene [26]. In addition, recent studies have shown strain engineering is still an efficient way to improve the transport properties of silicon nanowires [27]. Applying strain to a single layer of black phosphorus nanoribbon can also change the transport direction of carriers, which can control the anisotropy of carrier mobility [28]. Moreover, the strain can influence the spin characteristics of semiconductor. A valley polarization current can generate in graphene by adding the strain with respect to a raised bubbling structure [29].The strain-induced band convergence could be an effective method to enhance the thermoelectric performance of phosphorene [30]. Furthermore, the optical [31] and magnetic properties [32] of nanojunction can also be induced and modulated by strain. Thus, it is not difficult to see that the regulation of strain engineering on materials is valuable.

In recent years, carbon science has been widely affected the developing fields of molecular junctions [33–36]. By employing a cross-coupling reaction, Li et al*.* [37] have successfully synthesized graphdiyne sample on the surface of copper. Since then, graphdiyne has attracted great interest from international researchers [38, 39]. Graphyne is the allotrope of graphene with 2D plane network structure [14, 40–45], which is formed by the conjugation of benzene rings and the C–C link with the acetylenic bonds. Compared with the simple layered *sp*^2^ orbital hybrid structure of graphene [46], graphyne holds *sp* and *sp*^2^ hybridized states, determining that its unique molecular structure is more complicated. There are many existing members belonging to graphyne family, such as α-graphyne [40, 41], β-graphyne [47], γ-graphyne [42, 48, 49], α-2-graphyne [14], 6,6,12-graphyne [43], 14,14,14-graphyne [44], δ-graphyne [45] and so on. Among those exiting structures, γ-graphyne does not possess Dirac cone-like electronic structure around the Fermi level, which is quite different to graphene. Similar to graphene nanoribbons, γ-graphyne can also be cut into armchair and zigzag γ-graphyne nanoribbons (AγGYNs and ZγGYNs). Extensive works have exhibited on ZγGYNs to observe excellent performances, such as spin-filtering, negative difference resistance. However, the study of strain implemented on ZγGYN between gold electrodes has not been reported.

Motivated to explore the advantages of strain engineering on ZγGYNs, we introduce strain into the molecular junctions based on ZγGYN to carry out the research by using first-principles calculations. In this paper, we firstly concentrated on the electronic structures of ZγGYNs within different magnetic configurations. The observation displayed that the phenomenon of spin-splitting occurs after introducing strain into the junction, which imply that strain may be a means of manipulating spin. Further, the results on the spin-currents of junctions imply that the strain has an important influence on the transport properties of the device to some extent. And we find that the strain engineering can enhance the coupling between the electrode and intermediate scattering region, which widens the electronic channels.

## Models and Method

**Fig. 1:**
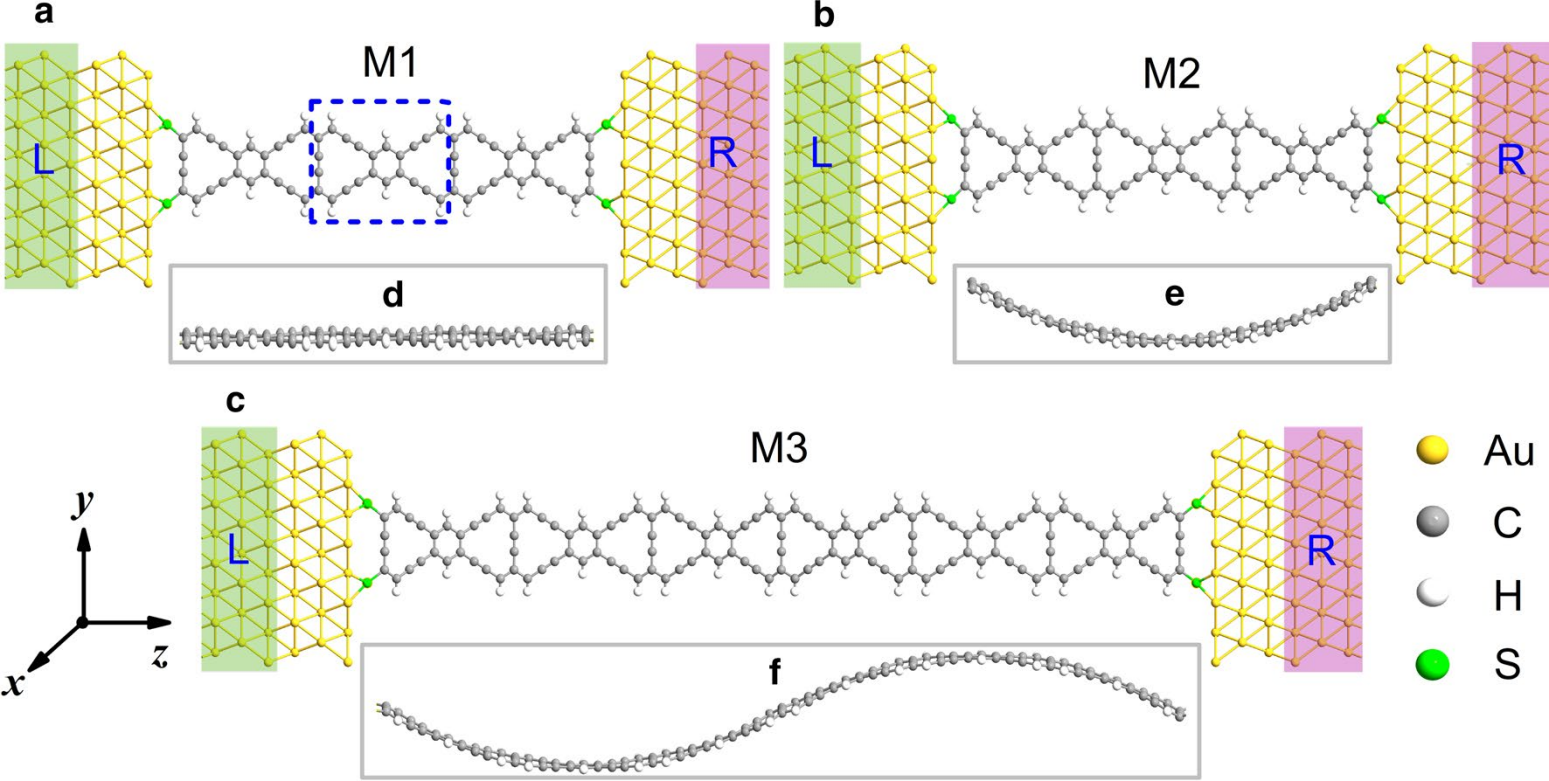
(Color online) Schematics of the molecular junction models has been displayed as **a** M1, **b** M2 and **c** M3, whose scattering region is flat, curved (U-shape) and double-curved (S-shape), respectively. The blue dashed rectangle in panel **a** donates the repeated unit cell of ZγGYNR, whose lattice constant is 12.297 Å. For clarity, the side-view in the scattering region for **d** M1, **e** M2 and **f** M3 corresponding to **a**–**c** have also been exhibited. L/R represents the left/right electrode

In Fig. 1, three different molecular junctions have been exhibited as M1, M2 and M3, respectively. The junctions can divide into three parts: left electrode, scattering region and right electrode. Here, we use the gold (Au) nanowire as the electrode material due to its good ductility and electrical conductivity. The Au electrode is cleaved on the (111) surface. And the scattering region is composed of several repeated ZγGYN units. The Au atoms of the leads and carbon (C) atoms in the central part are connected by sulfur atoms. For the experiment on the graphene junction, it is shown that the graphene nanoribbons can be tailored and cut into many structures as molecular devices in experiment by employing energetic electron irradiation inside a transmission electron microscope (TEM) [50]. Similar to graphene, the molecular devices based on ZγGYN perhaps can also be connected in this way. The M1 is not introduced with strain, and the scattering region is flat as shown in Fig. 1a. The M2 appears to be curved in the *x* axis with a *U*-curved structure which makes it no longer flat in Fig. 1b, which is resulted from the transverse strain. For the M3 system, whose structure is most complex, holds an *S*-curved structure. The original length of ZγGYN in the scattering region of M3 is twice larger than the one of M1. Thus, the ZγGYN with the strain effect can be bent into the opposite direction of + *x* and − *x* axis, making it present an *S*-curved structure in Fig. 1c. The side views of M1–M3 in Fig. 1e, f are correspond with the main views for the scattering parts in Fig. 1a–c. The detailed junctions can be seen from the following pictures.

We firstly optimize the designed unit cells and molecular structures by implementing the density functional theory calculation in the Atomistix ToolKit package [51, 52]. According to the results of optimization, the lattice constant of the unit cell is about 12.297 Å in Fig. 1a, and the length of scattering region for M1-M3 is about 36.891 Å, 35.473 Å and 70.559 Å in Fig. 1a–c. The bond length between gold and sulfur atom is 2.38 Å, and that between sulfur and carbon atom is 1.84 Å, 1.62 Å and 1.92 Å for M1-M3, respectively. The detailed computational parameters have been set as follows. The exchange-correction potential is employed as the generalized gradient approximation with Perdew–Burke–Ernzerh of functional [53]. The mesh cut-off energy for the electrostatic potentials is 150 Ry, and the temperature for Fermi function is set to 300 K. The force on each atom is smaller 0.02 eV/Å. In addition, a Monkhorst–Pack mesh of 1 × 1 × 100 is chosen, and the convergence criteria of electron density is 10^−5^ eV in total energy. Furthermore, in order to avoid the interaction between periodic images, at least 20 Å vacuum layer thickness is set in our calculations. The transmission spectrum as a function of energy (*E*) and bias voltage (*V*) is defined as

$$T_{\sigma } (E,V_{{\text{b}}} ) = Tr\left[ {\Gamma_{{\text{L}}} \left( E \right)G_{\sigma }^{{\text{R}}} \left( E \right)\Gamma_{{\text{R}}} (E)G_{\sigma }^{{\text{A}}} (E)} \right],$$

where $G^{{{\text{R}}({\text{A}})}}$ is the retarded (advanced) Green’s function of the central scattering area, $\Gamma_{{\text{L(R)}}}$ is the coupling matrix of the left (right) electrode and *σ* =  ± 1 donates the electron spin-up/down. The spin transport current is calculated by using the Landauer-Büttiker formula [54, 55]

$$I_{\sigma } \left( {V_{{\text{b}}} } \right) = \frac{e}{h}\int {T_{\sigma } \left( {E,V_{{\text{b}}} } \right)} \left[ {f_{L} \left( {E - \mu_{{\text{L}}} } \right) - f_{{\text{R}}} \left( {E - \mu_{{\text{R}}} } \right)} \right]{\text{d}}E,$$

where $\mu_{{\text{L(R)}}}$ and $f_{{\text{L(R)}}}$ are the electrochemical potential and the corresponding Fermi distribution function of the left/right electrode, respectively. The device density of states (DDOS) can be calculated by $D\left( E \right) = - \frac{1}{\pi }{\text{Im}} G^{{\text{R}}} (E)$.

## Results and Discussions

**Fig. 2:**
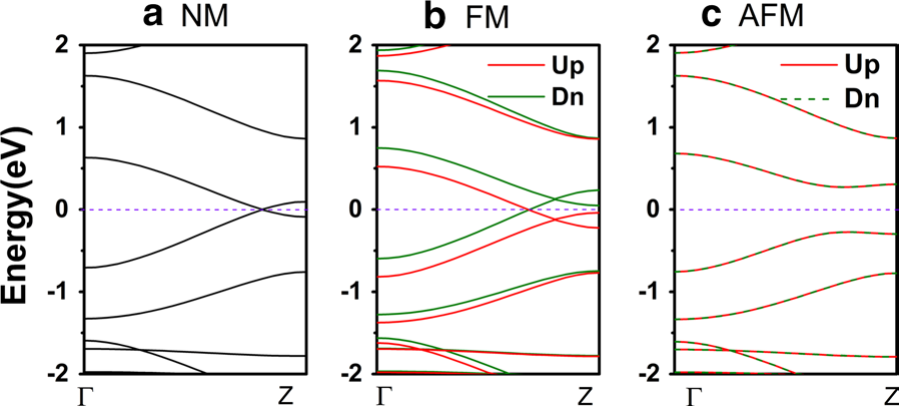
(Color online) The band structures for the ZγGYNR are shown within the **a** NM, **b** FM, and **c** AFM states, respectively. The Fermi level has been taken as zero

The band structures of zigzag γ-graphyne unit cells have been plotted within non-magnetic (NM), ferromagnetic (FM) and anti-ferromagnetic (AFM) states, as displayed in Fig. 2a–c, respectively. In the computational progress, the magnetism of the carbon atoms attached to the upper edge and lower edge is all set to the same direction, approaching to the FM state; the setting of AFM state is opposite. One can see that the ZγGYNR is metallic in NM state, in that the energy bands go through the Fermi level in Fig. 2a. Similar to the NM one, the ZγGYNR in FM state is also metallic, but the obvious spin-splitting can be observed. The energy band in spin-up direction is downshifted in Fig. 2b, while the spin-down band is upshifted. However, when the ZγGYNR is set in AFM state, the band structure exhibits a tiny band gap of 0.55 eV which makes it as a semiconductor in Fig. 2c. Furthermore, the corresponding total energies of the states have also been calculated for M1-M3, respectively. The relative results are displayed as follows: The energy of ZγGYNR unit cell in NM state is the highest of − 3524.42090 eV, and the one in AFM state is the lowest of − 3524.49299 eV. The energy difference between the highest and lowest energy is about 0.07 eV. Therefore, according to the data of all energies, we can draw the conclusion that the AFM state is the ground state of ZγGYNR. The FM state of ZγGYNR can induce the spin polarization of nanoribbon, and it would be applied in the field of spintronics. In the following, the deep transport mechanism for the three junctions have been expounded.

**Fig. 3:**
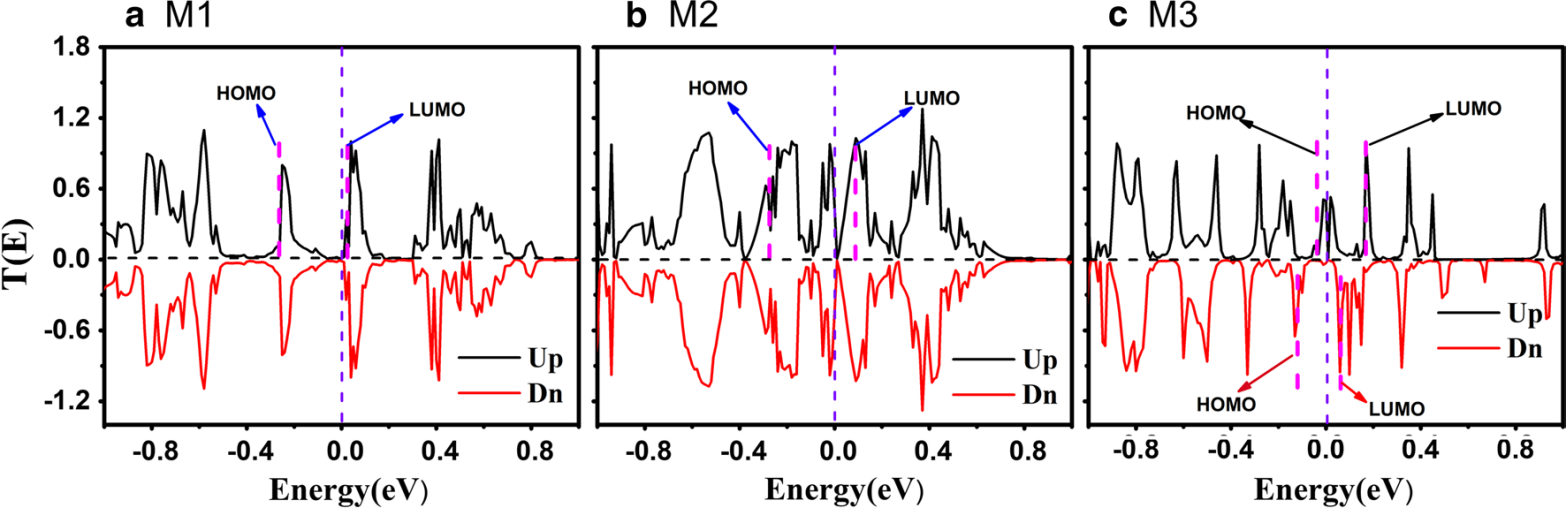
(Color online) The spin-dependent transmission spectra at zero bias have exhibited for **a** M1, **b** M2 and **c** M3, respectively. The spin-up and -down transmission coefficients have been set as the positive (black) and negative (red) values. Meanwhile, the distributions of molecular projected self-consistent Hamiltonian have been denoted here

Firstly, we plot the transmission spectra of the three junctions at zero bias in Fig. 3. There are many sharply pulsed peaks of the transmission spectrum and a tiny band gap near the Fermi level in Fig. 3a, suggesting that the M1 is a semiconductor. Thus, under the effect of suitable voltage, electrons can go through from the left to right electrode since the C=C or C≡C bond formed between carbon atoms provide a conductance channel for the electron transport. For the strained device of M2 in Fig. 3b, its transmission spectrum is not exactly the same as that of M1. There are still a lot of transmission peaks moving around the Fermi level. In other words, the transmission peaks of M2 with the effect of strain becomes wider than those of M1. In addition, the transmission peaks all seem to be getting closer to the Fermi level. This phenomenon generates from the effect of the strain on the scattering region of M2, which leads to the enhancement of the coupling between Au electrodes and intermediate ZγGYNR, making the transmission channels wider than that one of M1.

Further, in the case of M3, as shown in Fig. 3c, the most obvious feature is that the spin-splitting whose spin up (black solid line) and spin down (red solid line) transmission peaks are no longer degenerated. Moreover, the transmission peak of M3 is still as sharp as that of M1, but it becomes more dense as well. The spin up transmission peaks move forward the Fermi level, but the spin down transmission spectrum display a biggish transmission gap in Fig. 3c, resulting in that the M3 appears the spin-separated. This can be explained by the combination both the S-shape of M3 and the strain effect. The strain in the shape of S-shape changed the charge distribution of M3 and broke the original electric dipole, thus resulting in that the junction of M3 exhibits magnetic behavior and thus the spin-splitting phenomenon can be observed here. Obviously, the ZγGYNR for M3 is twice as long as that of M1, making the interaction between the electrodes and the scattering region weaker than M2. However, due to the asymmetric *S*-shape structure, the ZγGYNR is not in the same plane any more, for which could change the *sp* and *sp*^*2*^ hybrid components for γ-graphyne. Hence, the M3 is a more perfect model to design a new molecular junction.

**Fig. 4:**
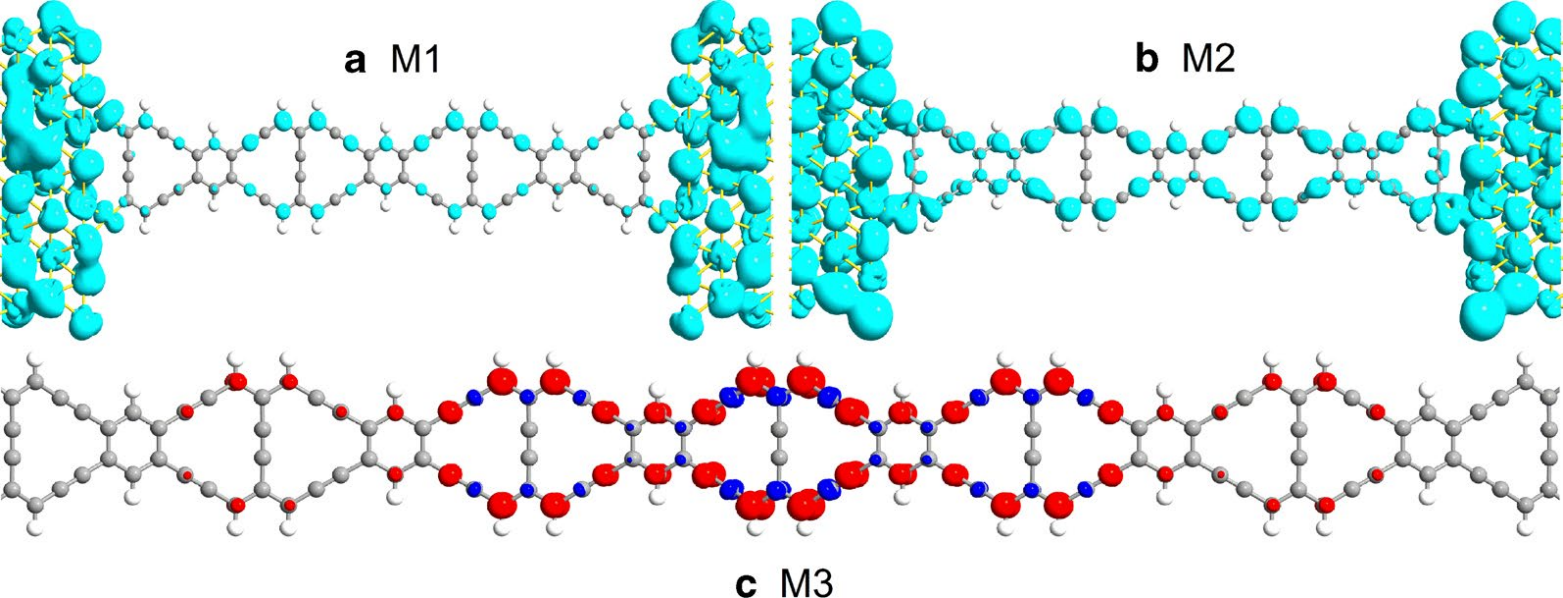
(Color online) The DLDOS at the energy of − 0.02 eV has been displayed as **a** M1 and **b** M2, respectively. The isovalue is taken as 0.01 Å^−3^·eV^−1^. **c** The spin density isosurface of M3 has also exhibited, where the red and blue colors stand for the spin-up and -down components, respectively. The isovalue is taken as 0.015 Å^−3^·eV^−1^

By carefully comparing the details of transmission peaks in Fig. 3a, b, it is of great importance to find that M1 has no transmission peak, and M2 has a very sharp peak at the energy of − 0.02 eV. To deeply understand the difference between M1 and M2, we draw the device local density of states (DLDOS) at − 0.02 eV, as shown in Fig. 4a, b. For M1 in Fig. 4a, it is worth to note that the electrons are primarily localized at the gold electrodes and the electron cloud lesser distribute in the area of ZγGYNR. Therefore, there are fewer transmission channels for charge transport for M1. But for M2, the electrons densely distribute in the electrodes and the scattered area of ZγGYNR throughout the whole ribbon, indicating that the rich transmission channels are provided for the electron transport, so the transmission spectra of M2 looks wider than M1 around the Fermi level. This result implies that the molecular junction of M2 with strain controlling would hold better transport property, and which will be discussed later.

**Fig. 5:**
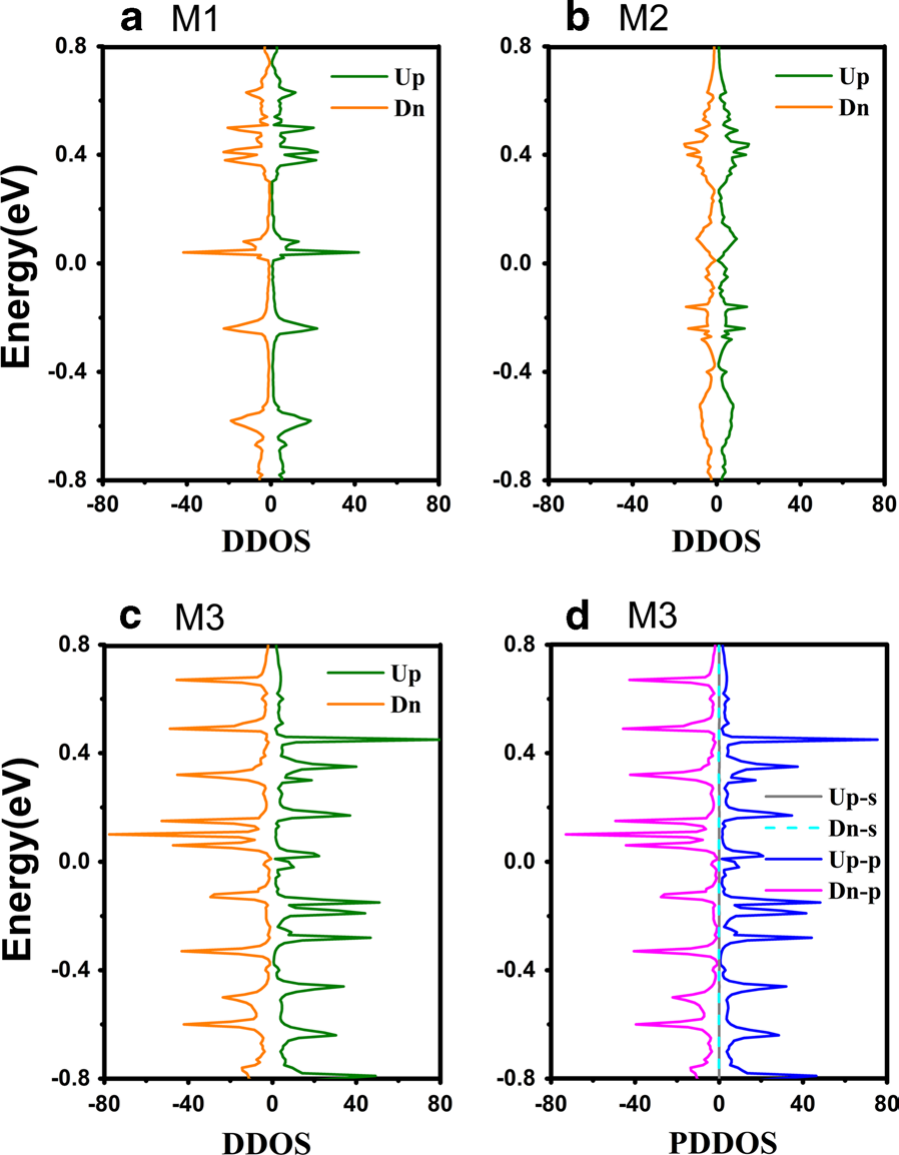
(Color online) The DDOS is displayed as **a** M1, **b** M2 and **c** M3, respectively. **d** is the projected device density of states (PDDOS) for M3. The "Up-s" and "Dn-s" stand for the *s-*orbital PDDOS in spin-up and -down direction, the "Up-p" and "Dn-p" stand for the *p-*orbital PDDOS in spin-up and -down direction, respectively

Furthermore, the corresponding DDOS for each model is given in Fig. 5a–c, where the green (orange) solid line represents the spin-up (-down) direction, respectively. Firstly, the shape and distribution of the DDOS in Fig. 5a–c are correspond to the transmission spectra as shown in Fig. 3a–c. The DDOS of M1 in Fig. 3a exhibits a sharp peak at *E* > 0, and the spin-up and spin–down DDOS are symmetric to the zero point. For M2 in Fig. 3b, the peaks of DDOS nearly extend to the whole Fermi level, contributing to the charge transport of molecular junction. Hence, the strain implemented in the M2 promote the peak move together in Fermi level. The similarity in the peak structure of the DDOS and the transmission spectrum indicates a clear correspondence between the energy levels of the ZγGYNR and the transmission spectra. The coupling between the Au electrodes and the ZγGYNR caused by strain greatly expands the transmission tunnel.

As seen from the transmission spectra and the DDOS, the spin-splitting phenomenon of M3 can also be observed in Fig. 5c, suggesting that the M3 with a long molecular chain of ZγGYNR is magnetic. In order to give an intuitive understanding on the magnetism of M3, the spin-density distribution is plotted in Fig. 4c, where the red and blue colors stand for the spin-up and spin-down components, respectively. It is notable to see that the atomic magnetic moments are mainly localized at the center of the nanoribbon and show a gradually weakening trend from the center to the edges in Fig. 4c. Similar to zigzag graphene nanoribbons, the ZγGYNRs are known to be magnetic [56]. However, due to the presence of strain, the coupling between the electrodes and the central region leads to the changes of original magnetic distribution. Thus, the magnetism of the atoms closest to the electrode disappears while the magnetism of the central region farthest from the electrode remains. To determine which orbitals are responsible for most of the magnetism, we plot the PDDOS of M3 in Fig. 5d. It is clear from the PDDOS that the *s-*orbital electrons hold little contribution to the magnetism of M3, since they trend to a zero value in the middle of the Fig. 5d. That is to say, the magnetism of M3 is mainly dependent on the *p-*orbital electrons since both the shape and position of the peaks are highly consistent with the DDOS in Fig. 5c. Therefore, the contribution of the outer electrons is much greater than that of the inner electrons in charge transport for M3. In order to display the transport properties for the M1-M3, the current–voltage (*I–V*) characteristics have been investigated in the following. The relative inner mechanism is revealed to verify the previous prediction.

**Fig. 6:**
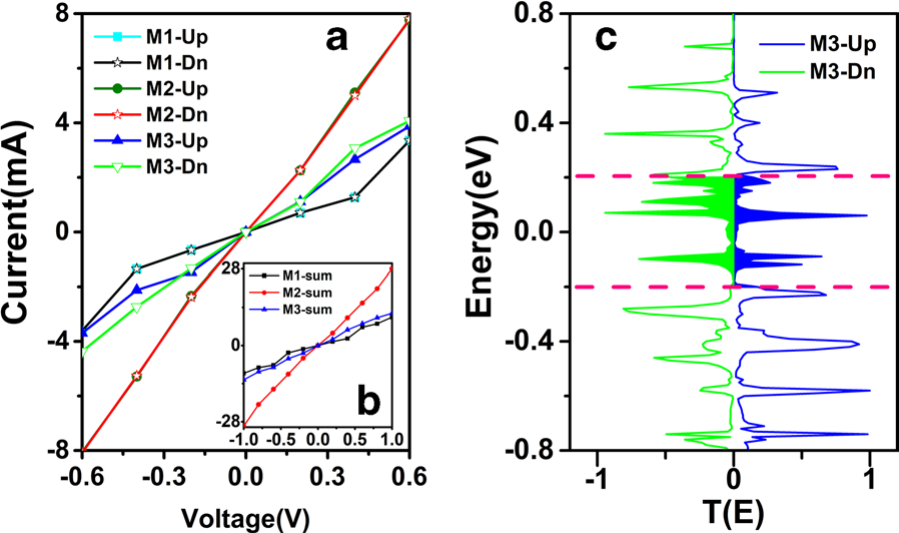
(Color online) **a** The calculated spin-dependent *I–V* curves as a function of the applied bias for M1, M2 and M3. The insert (a′) shows the calculated total currents for each device. **b** The spin-dependent transmission spectra for M3 at bias − 0.04 V. The shaded area between the pink dotted lines is the energy region contributing to the current, i.e. the bias window (The shaded blue and green area on the right and left sides donate the spin up and spin down, respectively.)

To further explore the corresponding mechanisms of the different performance for all systems, we calculate the *I–V* curves for the proposed M1–M3, as shown in Fig. 6a. Figure 6a is the calculated spin-dependent *I–V* curves as a function of the applied bias for each device and the insert Fig. 6a′ shows the calculated total currents. With the increasing of the voltage range from − 0.6 to 0.6 V, the current curve behaves symmetrically under positive and negative bias voltage ranges as shown in Fig. 6a. It is worth to note that the currents of M2 and M3 are obviously larger than that of M1, which demonstrates that the strain has a certain effect on the charge transport. The magnitude of current for M1 without strain is the smallest of the three junctions. It increases slowly as the bias voltage increasing. Furthermore, it is obviously seen that the current for M2 with a largest slope shows a rapid increase as the increasing of bias voltage. In particular, the current of M2 is almost three times than that one of M1 at the same bias voltage. On the contrast, the current for M3 with S-curved structure is moderate between M1 and M2, which shows a weaker conductive behavior than M2 but stronger than M1.

In addition, the spin-splitting phenomenon can also be found from the current for M3 in Fig. 6a. The *I–V* curve is quite consistent with the transmission spectra and DDOS mentioned above. There is no doubt that strain makes molecules no longer in the same plane, damaging the delocalized conjugate *π-*bond in the ZγGYNR. However, there is another aspect to consider, due to the squeezing effect, the coupling between the electrode and the scattering region is enhanced, so eventually the electron channel widens and the current increases. Thus, the current for M3 is also affected by strain, but not so much as that for M2. The following reasons can be explained. The length of scattering region reduces the coupling between the Au electrodes and the ZγGYNR to some extent, making the current of M3 only larger than M1 but smaller than M2. The strain effect and the length of ZγGYNR commonly determine the current intensity of M3. So, we can see that the spin-dependent current appears for M3 in Fig. 6a. This is also corresponding to the Fig. 5c. Although all the above calculation results show that the M3 with strain has spin-splitting phenomenon, it is indeed not very significant. For spin modulation there may be other more efficient means. In fact, some other methods, such as electric field [57, 58], edge modifications [59] and doping [60] can also induce spin-polarization and enhance spin-splitting in many two-dimensional based nano-devices.

From the results we know that M3 holds spin-splitting phenomenon, and it is not difficult to find that when the bias voltage is − 0.4 V, the difference in the current value of the spin up and spin down |*I*_Up_ – _Dn_| for M3 is the biggest, which can be seen from the blue and green solid lines in Fig. 6a. To this end, we plot the transmission spectra of M3 at -0.4 bias in Fig. 6b, in which the solid lines of blue and green donate the spin up and spin down components, respectively. We can see the transmission area of the green part in the bias window is bigger than that of the blue one, resulting in that the corresponding current of spin down is bigger than the spin up one at same bias voltage of − 0.4 V.

**Fig. 7:**
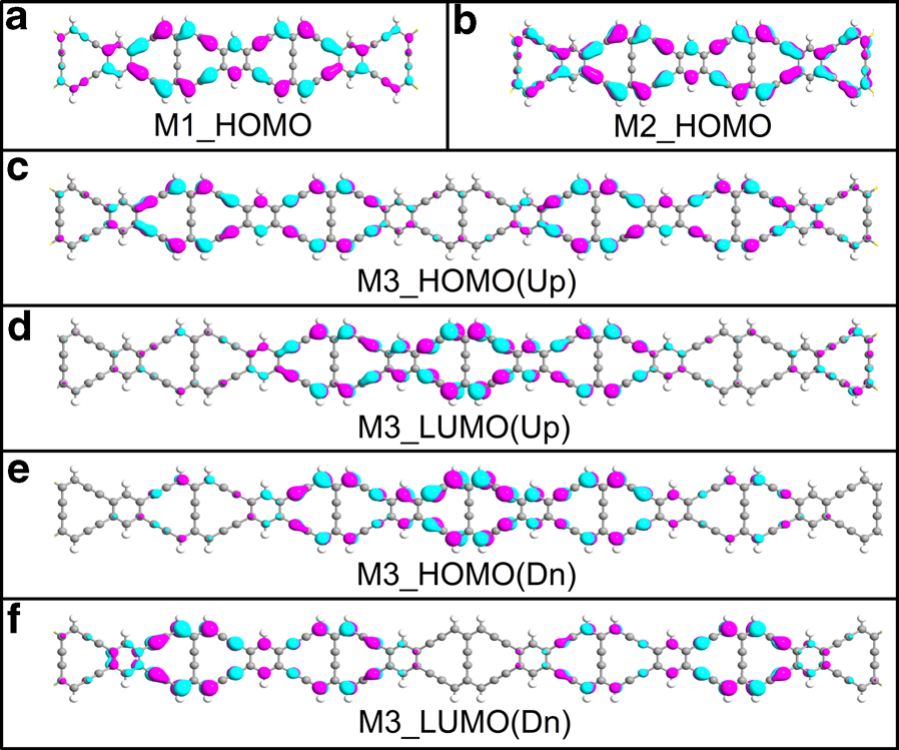
(Color online) The HOMOs for **a** M1 and **b** M2 in spin-up direction; **c**–**f** The spatial distribution of HOMOs and the LUMOs for M3 in different spin directions

With regard to the transmission spectrum in Fig. 3b, we know that the transmission peak of M2 near the Fermi level appears at − 0.02 eV, so the frontier molecular orbitals play major role in charge transport. In addition, the previous calculation results show that the currents of M1 and M2 are spin-independent, so the spatial distributions of molecular projected self-consistent Hamiltonian (MPSH) in spin-down direction for M1 and M2 are ignored here. The spatial distribution of the highest occupied molecular orbitals (HOMOs) for M1 in Fig. 7a is weaker than that one for M2 in Fig. 7b. One can see that the HOMO of M2 are well delocalized throughout the whole scattering region, resulting in that a largest current of M2 arises here. In the case of M3, the spin-up HOMO distributes in the double sides of ZγGYNR in Fig. 7c, while the lowest unoccupied molecular orbitals (LUMOs) is mainly localized in the central area in Fig. 7d. On contrary, due to the magnetism of the strained ZγGYNR, the wave function of HOMO in spin-down direction is localized in the central region in Fig. 7e, but the distribution of LUMO in Fig. 7f is similar to the one of HOMO in spin-up direction. The spatial distribution of MPSH is relatively localized at the certain region, indicating a smaller current for M3. In other words, the interaction of molecular orbitals depends on the combination between the complex and flexible atomic interaction and external effect.

## Conclusions

In summary, the electronic structures and the transport properties of strained junctions based on ZγGYNR have been studied and analyzed. Our results show that the AFM state of the designed ZγGYNR is the ground state, and the band structure in the FM state is spin-splitting. What is more, the strain has a vital effect on transport properties of molecular junction. At the same length, the strain greatly enhances the orbital coupling between the Au electrodes and the ZγGYNR. As a result, the electronic channels of M2 are widened, thus the electron transport behavior in M2 is much larger than that in M1. Furthermore, the length and direction of ZγGYNR still paly a certain influence on the transport characteristics of the junction. Specifically, the coupling between the Au electrodes and the ZγGYNR is weakened due to the increasing of length, so the current of M3 is smaller than that of M2. In addition, the magnetic distribution of M3 results in an obvious spin-splitting phenomenon. The corresponding mechanisms of transport properties are discussed in terms of the transmission spectra, the LDDOS and so forth. Our results may provide novel ideas for the next generation of flexible electronic devices in the further.

## Data Availability

The design of molecular junctions and computational calculations were carried out by Atomistix ToolKit package.

## Abbreviations

γ-GYNRs: γ-Graphyne nanoribbons
2D: Two-dimensional
NM: Non-magnetic
FM: Ferromagnetic
AFM: Anti-ferromagnetic
DDOS: Device density of states
DLDOS: Device local density of states
PDDOS: Projected device density of states
MPSH: Molecular projected self-consistent Hamiltonian
HOMO: Highest occupied molecular orbitals
LUMO: Lowest unoccupied molecular orbitals
Up: Spin-up
Dn: Spin-down
